# Glycosylated amyloid‐like proteins in the structural extracellular polymers of aerobic granular sludge enriched with ammonium‐oxidizing bacteria

**DOI:** 10.1002/mbo3.616

**Published:** 2018-03-31

**Authors:** Yuemei Lin, Clara Reino, Julián Carrera, Julio Pérez, Mark C. M. van Loosdrecht

**Affiliations:** ^1^ Department of Biotechnology Faculty of Applied Sciences Delft University of Technology Delft The Netherlands; ^2^ GENOCOV Research Group Department of Chemical, Biological and Environmental Engineering School of Engineering Universitat Autònoma de Barcelona Barcelona Spain

**Keywords:** aerobic granular sludge, ammonium‐oxidizing bacteria, amyloid, extracellular polymeric substances, glycoproteins

## Abstract

A new type of structural extracellular polymers (EPS) was extracted from aerobic granular sludge dominated by ammonium‐oxidizing bacteria. It was analyzed by Raman and FTIR spectroscopy to characterize specific amino acids and protein secondary structure, and by SDS‐PAGE with different stains to identify different glycoconjugates. Its intrinsic fluorescence was captured to visualize the location of the extracted EPS in the nitrifying granules, and its hydrogel‐forming property was studied by rheometry. The extracted EPS is abundant with cross ß‐sheet secondary structure, contains glycosylated proteins/polypeptides, and rich in tryptophan. It forms hydrogel with high mechanical strength. The extraction and discovery of glycosylated proteins and/or amyloids further shows that conventionally used extraction and characterization techniques are not adequate for the study of structural extracellular polymers in biofilms and/or granular sludge. Confirming amyloids secondary structure in such a complex sample is challengeable due to the possibility of amyloids glycosylation and self‐assembly. A new definition of extracellular polymers components which includes glycosylated proteins and a better approach to studying them is required to stimulate biofilm research.

## 1 INTRODUCTION

Aerobic granular sludge (AGS) process has evolved in the last two decades as an alternative to conventional activated sludge wastewater treatment (Pronk et al., 2015). In AGS, microorganisms produce a significant amount of highly hydrated extracellular polymers (EPS) to form a structural hydrogel matrix in which they are self‐immobilized (Sam & Dulekgurgen, 2016; Seviour, Pijuan, Nicholson, Keller, & Yuan, 2009). The hydrogel‐forming polymers are referred as “structural EPS” (Felz, Al‐Zuhairy, Aarstad, van Loosdrecht, & Lin, 2016), to distinguish them from other EPS compounds. In order to extract the structural EPS from aerobic granular sludge, solubilizing the hydrogel matrix is the necessary first step (Felz et al., 2016; Pronk, Neu, van Loosdrecht, & Lin, 2017). Alkaline or acidic conditions are currently used. For example, Na_2_CO_3_ was used to solubilize the AGS fed municipal wastewater resulting to alginate‐like extracellular polymers (ALE) extracted as the structural EPS (Lin, de Kreuk, van Loosdrecht, & Adin, 2010). NaOH was used to solubilize the AGS fed with anaerobically treated abattoir wastewater with supplementary acetate dosing resulting to Granulan was extracted as a gel‐forming polymer (Seviour, Lambert, Pijuan, & Yuan, 2010). Hot acetic acid was needed to solubilize the AGS fed with sodium acetate at 35°C, and an acid soluble EPS with O‐methyl glucose and galactose as monomers was extracted as the structural EPS (Pronk et al., 2017). These first observations indicate that different AGS might have different structural EPS.

Recently it was reported that AGS highly enriched with ammonia‐oxidizing bacteria (AOB) performed partial nitritation at 10°C for more than 8 months. The ammonium oxidation rate was extremely high at such low temperature (ca. 0.6 g·N·L^−1^·d^−1^ at 10°C) (Reino, Suárez‐Ojeda, Pérez, & Carrera, 2016). Interestingly, no EPS could be extracted from these granules when the protocols of ALE (Lin et al., 2010), Granulan (Seviour et al., 2010) and acidic soluble EPS (Pronk et al., 2017) extraction were followed. The AGS was not solubilized using any of these protocols. Apparently, the three reported extracellular polymers are not the structural EPS of the AOB dominated granules. Therefore, a new type of polymers is likely present as the structural EPS in this kind of granular sludge.

The aim of this research was to extract this new type of extracellular polymers from granular sludge enriched with AOB, analyze its chemical structure and unravel its role in maintaining the granular structure. After the structural EPS was extracted, Raman and FTIR spectroscopy were used to characterize specific amino acids and protein secondary structure, SDS‐PAGE with different stains was employed to identify the presence of different glycoconjugates separately. Intrinsic fluorescence was captured to visualize the location of the extracted EPS in granules and rheological property was studied to understand EPS hydrogel formation. Obstacles of conventional EPS extraction and characterization to the study of structural EPS in biofilm are pointed out and discussed.

## 2 EXPERIMENTAL PROCEDURES

### 2.1 Aerobic granular sludge and the dominant microorganisms

Aerobic granular sludge enriched with AOB was cultivated in a lab‐scale airlift reactor performing stable partial nitritation of a low‐strength synthetic wastewater for more than 2 years. The granular airlift reactor operated at temperatures ranging from 30 to 10°C, being operated at 10°C during the last 250 days before the sample for EPS characterization was taken (Isanta, Reino, Carrera, & Pérez, 2015; Reino et al., 2016). Details of reactor operation, synthetic wastewater composition are included in the Data S1.

The granular sludge was collected from the reactor, fixed by following the method described in Reino et al. (2016), and observed using a scanning electron microscope (EVO MA10; Zeiss, Germany) at the following conditions: 20 kv, 100 pA, secondary electron detector (SE1).

Abundances of AOB and nitrite‐oxidizing bacteria (NOB) were analyzed by FISH coupled to confocal laser scanning microscopy (CLSM) according to Reino et al. (2016) (Data S1).

### 2.2 Structural EPS extraction from granular sludge

The EPS of the granular sludge was extracted by following different methods that were reported in literature (Liu and Fang, 2002, Felz et al., 2016; Seviour et al., 2009; Pronk et al., 2017).

In addition, granules (1 g) were put into 300 ml 0.1% (w/v) sodium dodecyl sulfate at 100°C for 30 min while being stirred with a magnetic stirrer at 400 rpm, the pH was kept at 9 with the addition of 0.1 mol/L NaOH. After centrifugation at 1968 × *g* for 20 min at 4°C, the pellet was discarded. Polymers in the supernatant were precipitated out by adding 100 ml ethanol, 0.01 g of the extracted EPS was used for autofluorescence check, whereas the rest was lyophilized.

### 2.3 Characterization of the extracted extracellular polymeric substances

#### 2.3.1 Contents of proteins and carbohydrates

The carbohydrates content of the extracted EPS was measured as glucose equivalents using the phenol‐sulfuric acid assay (Dubois et al., 1956). The proteins content was measured as BSA (Bovine Serum Albumin) equivalents using the bicinchoninic acid (BCA) method (Interchim Uptima BC assay quantitation kit).

#### 2.3.2 Chemical structure analysis by Raman spectroscopy and Fourier transform infrared spectroscopy (FTIR)

Raman spectra of the lyophilized EPS over the spectral range 100–3,000 cm^−1^ were obtained with a dispersive‐type Renishaw micro spectrometer, InVia Reflex model, equipped with a CCD detector. Leica microscope with objective magnification x50 was used to focus the laser beam on the sample placed on a X‐Y motorized sample stage. Excitation source was provided by a 785 nm diode laser. Near‐infrared illumination was chosen to reduce sample intrinsic fluorescence. The spectrum was recorded with 50% laser intensity on the sample, with 5 accumulation using 10 s exposure time.

The Fourier transform infrared spectrum of the lyophilized EPS and granules were recorded on a FTIR Spectrometer (Perkin Elmer, Shelton, USA) equipped with a DTGS Mid‐infrared detector and a Golden Gate single reflection diamond attenuated total reflectance (ATR) cell. Spectra were recorded from 600–4,000 cm^−1^ at room temperature. The absorbance of the samples and background were measured as 64 scans each. Spectral analyses were performed with OriginPro^®^(OriginLab, US) and PeakFit^®^ (Systat Software Inc., US). These include calculation of the second derivative spectra using OriginPro^®^ (without any smoothing) and curve‐fitting of the amide I absorption spectra using PeakFit^®^. The Fitting of the amide I absorption spectra was performed between 1,700 and 1,600 cm^−1^ and carried out using nonlinear least squares. The frequencies of the band centers found in the second‐derivative spectra in the amide I regions were used for Gaussian curve fitting and the half width of each band was set within 10–30 cm^−1^. The areas of the individual assigned bands and their fraction of the total area in the amide I region are calculated to quantify protein secondary structures.

#### 2.3.3 Protein analysis by SDS‐PAGE (Sodium dodecyl sulfate – polyacrylamide gel electrophoresis)

The SDS‐PAGE was performed using NuPage^®^ Novex 4%–12% Bis‐Tris gels (Invitrogen) according to the Invitrogen NuPAGE^®^ specifications. In brief, 10 mg of the lyophilized EPS was dissolved in 4 ml of 50 mmol/L Tris‐buffer as a stock solution. 6.5 μl of this stock solution were mixed with 2.5 μl of 4 × LDS sample loading buffer (Invitrogen) and 1 μl of 0.1 mol/L dithiothreitol (DTT) and heated at 70°C for 10 min EPS samples. Subsequently, 10 μl (16 μg EPS) sample was loaded per well. Electrophoresis was performed at room temperature for 35 min using a constant voltage (200v) in 1 × solution of NuPAGE MES SDS running buffer (Invitrogen). The gels were stained by three different stains afterwards: Colloidal Blue staining (Coomassie brilliant blue, Invitrogen); periodic acid‐Schiff (PAS) staining (Thermo Scientific Pierce Glycoprotein Staining Kit); and Alcian Blue 8GX (Fluka, Sigma Aldrich) (Table 1).

**Table 1:** Stains used to detect carbohydrates and proteins of the extracted extracellular polymers in SDS‐PAGE (Arellano, Storch, & Sarasquete, 2001)

Stains	Function and/or components demonstrated
Coomassie brilliant blue	Proteins in general
Periodic acid‐Schiff (PAS)	Vicinal hydroxyl groups in glycoconjugates
Alcian Blue pH 2.5	Carboxyl‐rich glycoconjugates and/or Sulfated glycoconjugates
Alcian Blue pH1.0	Sulfated glycoconjugates

To check if further denature of proteins improves SDS‐PAGE analysis, EPS was dissolved in 50 mmol/L Tris‐buffer to achieve a concentration of 5 mg EPS/ml as a stock solution (A). The stock solution A (15 μl) was mixed with 25 μl of 4 × LDS sample loading buffer (Invitrogen) as stock solution B. Stock solution B (4 μl) was mixed with 5 μl of reducing buffer (2M thiourea, 8M urea, and 3% SDS), 1 μl of 0.1M DTT and subsequently boiled at 100°C for 10, 30, and 60 min. Samples were run on NuPage^®^ Novex 4%–12% Bis‐Tris gels and stained by three different stains as described above.

#### 2.3.4 Intrinsic fluorescence of the EPS and the granules

The auto fluorescence of both the extracted EPS before lyophilizing and the fresh granules were microscopically examined with a Leica TCS‐SP5 confocal laser scanning microscope (Leica Micro‐system Heidelberg GmbH; Mannheim, Germany).

#### 2.3.5 Rheological property of the EPS and the granules

The temperature of the tested samples was maintained at 25°C. The viscoelastic response of aqueous EPS and the fresh granules was examined by carrying out oscillatory measurements (including strain sweep and frequency sweep tests) on an Anton Paar, Physica MCR 501 modular rheometer provided with a Peltier system for temperature control. A solvent trap was used to avoid solvent evaporation. All measurements were carried out in parallel‐plate geometry with a diameter of 50 mm, and a gap of 1 mm. The shear stress (strain) amplitude was changing in order to determine the limit of linear viscoelastic response. Hence, the mechanical spectra in the range of the linear viscoelastic regime were obtained with frequency sweeps at a constant strain amplitude of 0.5%. Every frequency sweep given in this research displays values that are an average of three measurements.

## 3 RESULTS

### 3.1 Nitrifying aerobic granular sludge and the dominant microorganisms

**Figure 1:**
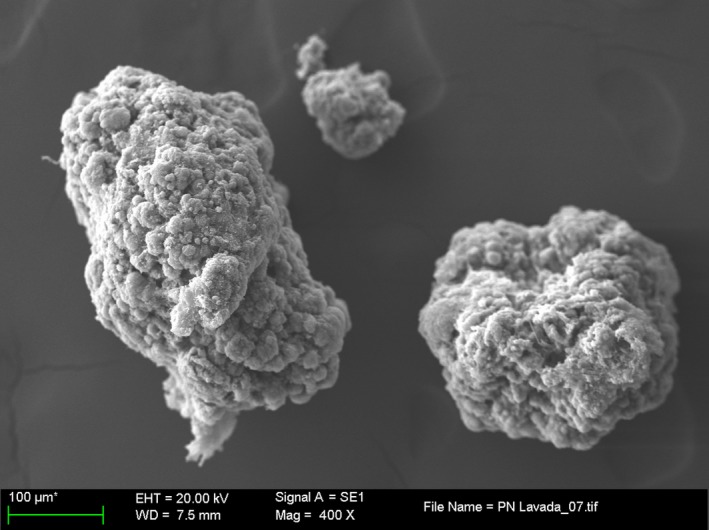
Scanning electron microscopy (SEM) image of nitrifying aerobic granular sludge collected from the reactor

Typical morphology of the nitrifying aerobic granular sludge was observed with scanning electron microscopy as shown in Figure 1. The average size of the granules in the reactor was around 800 μm. The surface of granules shows spherical‐shaped microcolonies around 10–20 μm in diameter.

**Figure 2:**
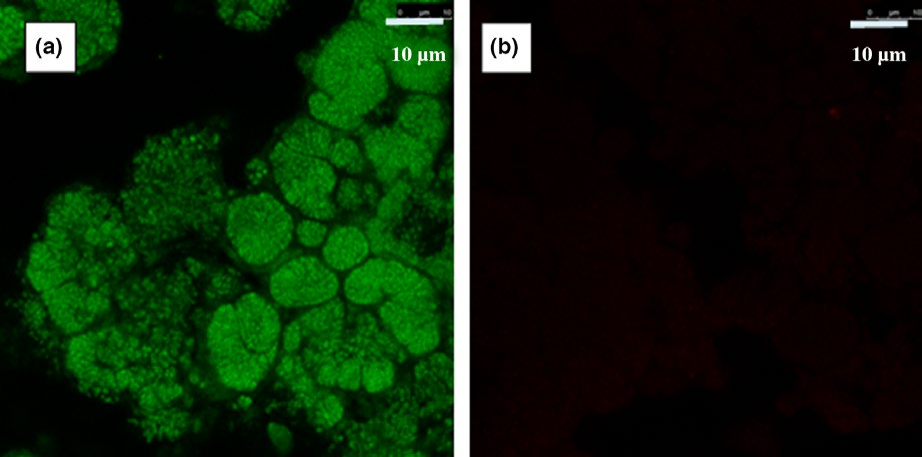
Dominance of ammonium‐ oxidizing bacteria in nitrifying aerobic granular sludge analyzed by fluorescence in situ hybridization. (a) epifluorescence image of granular sludge hybridized by AOB probe (b) epifluorescence image of granular sludge hybridized by NOB probe

The nitrifying aerobic granular sludge was highly enriched in AOB during the whole operation of the airlift reactor. When the samples were collected, the abundance of AOB was 92% ± 4% of the total population as quantified by fluorescence in situ hybridization (FISH) coupled to confocal laser scanning microscope as described in Isanta et al. (2015) and Reino et al. (2016) (Figure 2a). Regarding NOB, *Nitrobacter* spp. were quantified as 1% ± 1% of the total population (Figure 2b). Neither *Nitrospira* spp. nor *Nitrosospira* spp. (NOB) were detected in the sludge sample. Thus, most of microcolonies observed by SEM are the microcolonies of AOBs.

### 3.2 Structural EPS extraction

Normally AOB cells grow in microcolonies embedded in EPS (Larsen, Nielsen, Otzen, & Nielsen, 2008; Larsen, Nielsen, Svendsen, & Nielsen, 2008). To extract the structural EPS nitrifying granules dominated by AOB, various physical and chemical methods described in literature were used: centrifugation, sonication, use of cation exchange resins, addition of EDTA, NaOH, sodium carbonate, and acetic acid. None of these methods solubilized the granular sludge, that is, the granular structure was still intact after all these extraction trials, implying no structural EPS were extracted from the granules by these methods.

**Figure 3:**
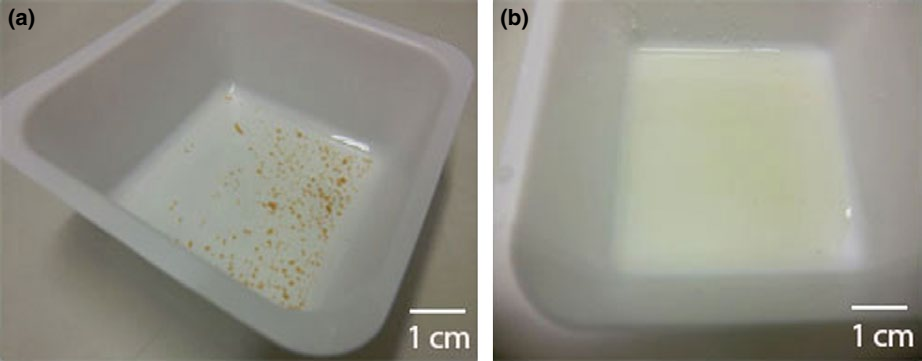
Nitrifying granular sludge before and after SDS extraction. (a) Granules before extraction. (b) Absence of granular structures after heating in 0.1% SDS extraction at pH 9 and 100°C for 30 min

When seeking more effective methods to solubilize the granular structure, it was observed that, after the granules were heated in 0.1% SDS at pH 9 and 100°C for 30 min, the granular structure disappeared. Instead, the nitrifying granules changed from solid into a solution‐like liquid, indicating the matrix of granules was totally solubilized (Figure 3). With this extraction protocol, 0.48 ± 0.09 g organic matter g^−1^ granules (VSS ratio) was recovered.

### 3.3 Characterization of the structural EPS

#### 3.3.1 Protein quantification and tryptophan content

In the extracted EPS, the content of proteinaceous material was measured as 2 g BSA‐equivalents g^−1^ EPS, while the content of carbohydrate material was measured as 19 mg glucose‐equivalents g^−1^ EPS. Apparently, the measured proteinaceous content was extremely abnormal, that is, it seemed twice the weight of the EPS itself. The tests were repeated several times with different dilutions of the EPS, but similar results were obtained each time.

**Figure 4:**
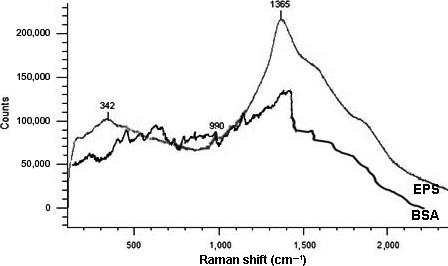
Raman spectrum of the extracted extracellular polymers (EPS) and Bovine Serum Albumin (BSA)

To understand the cause of the overestimation of the protein amount, Raman spectroscopy of the EPS was performed to get more structural and chemical information. There is a significantly intense peak at 1,365 cm^−1^ (Figure 4), which is a typical peak of the indole ring of tryptophan (Krafft, Hinrichs, Orth, Saenger, & Welfle, 1998). Comparing to the Raman spectrum of BSA, which does not have this intense peak at 1,365 cm^−1^, it is evident that the amount of tryptophan in the EPS is much higher than in BSA (0.59 g tryptophan 100 g^−1^ BSA) (Miura, Takeuchi, & Harada, 1988; Sparhr & Edsall, 1964). This higher amount of tryptophan could be one of the reasons for the overestimation of the protein content: in the current research, Bicinchoninic acid (BCA) assay was used to measure the amount of proteinaceous material. In this assay only amino acids such as cysteine, cystine, tryptophan, and tyrosine are detected (Smith et al., 1985; Wiechelman, Braun, & Fitzpatrick, 1988). In addition, there is always a reference standard used (in most cases BSA). The result of this assay is not the actual protein mass concentrations in the samples, but the equivalent amount of BSA standard that gives the same absorbance (Le, Kunacheva, & Stuckey, 2016). If the extracted EPS has higher amount of tryptophan than the BSA standard, the absorbance of the reaction might be higher and results to an overestimated amount of protein content.

#### 3.3.2 Protein secondary structure

Despite the overestimation of protein content, the extracted EPS seems to have a relatively higher mass of proteins than of carbohydrates. The secondary structure of proteins (α‐helices, β‐sheets, β‐turn, and random coil) is of crucial importance to their stability (Pirovano & Heringa, 2010) and mechanical properties. Therefore, FTIR spectroscopy was used to determine the secondary structure of EPS proteins.

**Figure 5:**
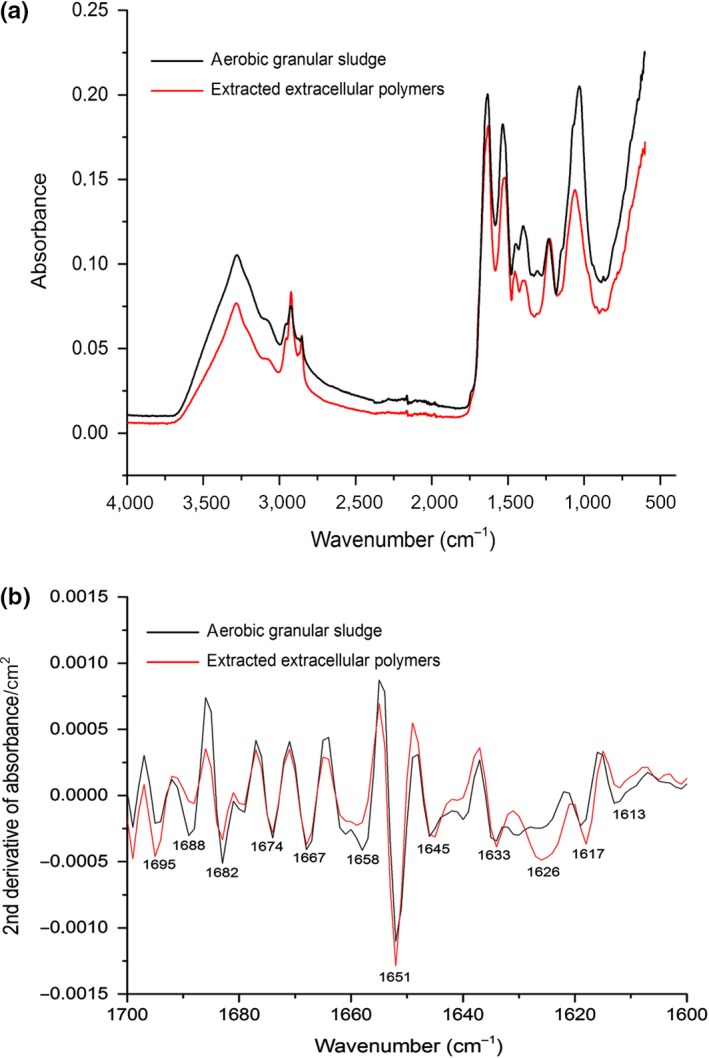
FTIR spectrum of nitrifying aerobic granular sludge and the extracted EPS. (a) FTIR spectrum; (b) secondary derivative of amide I region

As shown in Figure 5a, the FTIR spectra of the extracted EPS and the nitrifying aerobic granular sludge are extremely similar, indicating the extracted EPS is the dominant material in granules. The broad shoulder band around 3,050 cm^−1^ is assigned to aromatic CH from aromatic amino acids (tryptophan, tyrosine, and phenylalanine) (Dian et al., 2002). High amount of tryptophan found by Raman spectroscopy contributes to the appearance of this band.

The amide I region (1,700–1,600 cm^−1^) has been widely used to quantify the secondary structural composition of proteins and polypeptides (Yang, Yang, Kong, Dong, & Yu, 2015). Each type of secondary structure gives rise to a different C=O stretching frequency in this region. To increase the apparent resolution of overlapping bands, second derivative spectra of the amide I region were examined. The second‐derivative spectra of both EPS and the nitrifying aerogic granular sludge are significantly similar as well, implying that the extraction with 0.1% SDS at 100°C did not change the secondary structure of proteins at all (Figure 5b).

Secondary structures, α‐helices, β‐sheets, β‐turn, and random coil, were assigned according to values reported elsewhere in the literature (Barth, 2007; Barth & Zscherp, 2002). Although there is a slight difference in frequency assignment in literature, briefly, signals between 1,641 and 1,620 cm^−1^ and 1,695–1,680 cm^−1^ were assigned to β–sheets. Signals between 1,680 and 1,660 cm^−1^ were assigned to β–turns or turn‐like structures, signals between 1,660 and 1,650 cm^−1^ to α‐helices and between 1,650 and 1,641 cm^−1^ to random coil structures. The vibration frequency of each band, the assignment to the secondary structures and the percentage distribution are listed in Table 2.

**Table 2:** Amide I band frequencies, assignments to secondary structures and percentage distributions of the proteins in the extracted extracellular polymers

Mean frequencies (cm^−1^)	Assignment	Mean percentage (%)
1,617 ± 2	β ‐sheet	8.9
1,626 ± 1	Cross‐β‐sheet	22
1,633 ± 2	β‐sheet	22
1,645 ± 2	Random coil	13.1
1,651 ± 1	α‐helix	24
1,667 ± 2	β‐turn	2.7
1,674 ± 2	β‐turn	2.1
1,685 ± 3	β‐turn	0.99
1,693 ± 2	β‐sheet	4

The proteins in EPS contains about 57% of β –sheets, 24% of α‐helix, 13% of random coil and 6% of β‐turns. It is worth pointing out that the bands from 1,630 cm^−1^ to 1,611 cm^−1^ are specifically assigned to cross β‐sheet structure (Nielsen, 2010; Zandomeneghi, Krebs, McCammon, & Fändrich, 2004). This structure is a common feature of amyloids, which are defined as fibrillar polypeptide aggregate with a cross‐β structure (Fandrich, 2007). Amyloids have been intensively studied in the medical field and are hallmarks of many human illnesses including Alzheimer's, Parkinson's, and Huntington's diseases (Breydo, Wu, & Uversky, 2012). More recently, a new class of amyloids, “functional amyloids” have been described, to distinguish them from their disease‐related counterparts. Numerous functional amyloids are produced by a variety of microbes (Blanco, Evans, Smith, Badtke, & Chapman, 2012). According to Nilsson (2004), there is currently no amyloid criterion for the amount of cross β‐sheets secondary structure necessary to qualify a sample containing amyloid fibrils, that is, any percentage is acceptable. Therefore, the 22% cross β‐sheets contained in the EPS indicates the presence of amyloids.

In summary, the extracted EPS is protein rich and has cross β‐sheet structure which is a typical feature of amyloids.

#### 3.3.3 Denaturation resistance property and glycoconjugates

**Figure 6:**
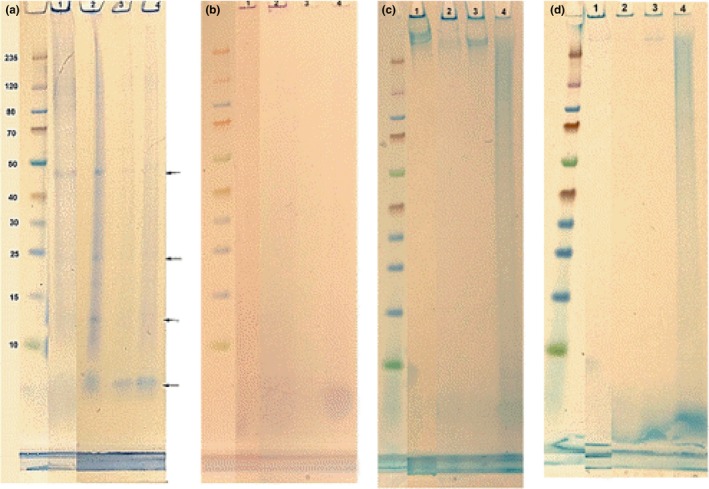
SDS‐PAGE analysis of the extracted extracellular polymers. (a) Coomassie blue stain; (b) PAS stain; (c) Alcian blue stain (pH 2.5); (d) Alcian blue stain (pH 1). Lane 1: the extracted EPS without any pretreatment; Lane 2: the extracted EPS with the pretreatment by heating at 100°C in (8M urea+3% SDS+2M thiourea) for 10 min; Lane 3: the extracted EPS with the pretreatment by heating at 100°C in (8M urea + 3% SDS+2M thiourea) for 30 min; Lane 4: the extracted EPS with the pretreatment by heating at 100°C in (8M urea + 3% SDS + 2M thiourea) for 60 min

A diagnostic feature of most amyloid aggregates is their resistance to denaturation in the presence of SDS, consequently remaining in the wells of SDS‐PAGE gel (Sivanathan & Hochschild, 2012). As the EPS is rich in cross β‐sheets secondary structure, it was analyzed by SDS‐PAGE to see if a similar feature was observed. Coomassie blue was used to stain proteins/polypeptides. It is shown by this staining that, besides three weak bands at around 48, 24, and 12 kDa, most of the EPS stays in the well (Figure 6a), meaning the majority of EPS is SDS resistant, still form aggregates with the size larger than the pore size of the gel. The EPS in the well was also strongly stained by PAS and Alcian blue (both at pH 2.5 and 1) (Figure 6b–d), implying the presence of glycoconjugates with vicinal hydroxyl groups, carboxyl groups (–COO^−^), and sulfate ester groups (–O–SO_3_^−^). In addition, there is a band at 400–235 kDa which was strongly stained by alcian blue at pH 2.5, very weakly by alcian blue at pH1 and not stained by the other two stains. This could be glycoconjugates rich in carboxyl groups, whether these glycoconjugates linking with proteins needs further investigation. This is because Coomassie blue has an overall charge of −1 at pH of 2 up to the neutral pH (Technical Info, 2010). Carboxyl groups are negatively charged at this pH range as well, they may inhibit the penetration of Coomassie blue to bind the protein core in glycoproteins.

According to literature, adding urea, SDS and heating the sample counters the phenomenon of amyloids “staying in the well” by further denaturing the peptide and preventing aggregation (Dueholm & Nielsen, 2016; Pryor, Moss, & Hestekin, 2012). It was observed that, after the EPS was treated with 2M thiourea + 8M urea + 3% SDS at 100°C for 10 min, it was indeed partially disaggregated, showing a Coomassie blue‐positive long smear with enhanced bands at 48, 24, and 12 kDa. Besides, a band with the molecular weight lower than 10 kDa (it was estimated as 4–6 kDa) appeared. Those changes imply that the pretreatment caused part of protein aggregates to disassemble into smaller fragments. It was reported that Amyloid‐β (Aβ_1‐42_) monomer is 4.5 kDa (Pryor et al., 2012). When SDS‐PAGE was used to separate oligomers formed by Amyloid‐β, it revealed bands for monomer (4.5 kDa), trimer/tetramer (16.5 kDa), and higher molecular weight intermediates (>83 kDa) that appeared as a smear (Zhang et al., 2009). If the 4–6 kDa band in this research is considered as a monomer, the other bands could be dimers/trimer (12 kDa), tetramers‐ hexamers (24 kDa), and octamers‐decamer (48 kDa). In comparison, full 70‐min incubation at 95°C with SDS concentration varying from 0.5% to 7.5% (w/v) is required for the partial or complete breakage of amyloid aggregates into oligomers (Bagriantsev & Liebman, 2004). The long smear observed in the current research could be oligomers. As many functional amyloids are highly adhesive and easily self‐assemble from their monomeric counterparts (Dueholm & Nielsen, 2016), the oligomers form smears in the SDS‐PAGE due to their continuous associations and disassociations, and attachment and detachment during the electrophoresis analysis (Pryor et al., 2012).

After the EPS was incubated with 2M thiourea + 8M urea + 3% SDS at 100°C for 30 min, the intensity of the Coomassie blue‐positive smear decreased. Bands at 48, 24, and 12 were faded and the band at 4–6 kDa had become narrower and intense, implying those oligomers were disassembled into monomers after 30 min (Figure 6a).

When the incubation time was increased to 60 min, the band at molecular weight 400–235 kDa disappeared, instead, there was a long smear through the whole lane. It was heavily stained by Alcian blue (pH 2.5 and 1), and weakly stained by Coomassie blue, implying they are glycoproteins carrying carbohydrate side chains with a few carboxyl groups and sulfate ester groups. This long smear is possibly due to further disaggregation of the fraction at 400–235 kDa into smaller oligomer fragments. Those oligomer fragments were more easily stained by Coomassie blue and Alcian blue than the polymer. On the other hand, since carbohydrates are usually heterogeneous and displaying a broad range of molecular weight distribution, this property may result to the relevant glycoproteins having a wide distribution of molecular weight, showing a smear on the SDS‐PAGE gel as well. The other interesting phenomenon was that the intensity of the Coomassie blue‐positive band at 4–6 kDa was increased. The lower part of this band overlaps with short smear which was positively stained by PAS and Alcian blue (pH 2.5 and 1), indicating that at least part of the monomers are glycopolypeptides, with vicinal hydroxyl groups, carboxyl groups, and sulfate ester groups at the carbohydrate part. Those carbohydrate groups probably locate on the polypeptide surface. Due to the negative charge of the carboxyl groups and sulfate ester groups, they oriented toward the positive pole (the bottom of the gel). Thus, they appeared at the lower part of the polypeptide band. Coincidentally, the monomers released at pretreatment times of 10 and 60 min appeared at the same position with Coomassie blue staining, despite the fact that the former was hardly glycosylated. Whether those are the same polypeptides with the only difference in glycosylation and if both of them have cross‐ β sheet structure need further investigation.

It was noticed that, after all these treatment, there was still EPS in the well which were positively stained by Coomassie‐blue, PAS and Alcian blue (pH 2.5 and 1), showing they are large complexes and extremely stable. Analysis other than polyacrylamide gels will have to be used to analyze them.

Glycosylated proteins/polypeptides with vicinal hydroxyl groups, carboxyl, and sulfate ester groups were found in the extracted EPS. The presence of protein glycosylation in prokaryotes has been demonstrated and accepted only recently (Szymanske & Wren, 2005). Most prokaryotic glycoproteins research focuses on S‐layers, pilins, and flagellins plus a selection of cell surface and secreted proteins which are known to be involved in adhesion and or biofilm formation (Dell, Galadari, Castre, & Hitchen, 2010). No information concerning glycoproteins in the EPS of aerobic granular sludge could be found in literature. In the amyloid research field, amyloid glycosylation has not been thoroughly investigated neither, although there are highly intriguing correlations between protein glycosylation and Alzheimer disease (Schedin‐Weiss, Winblad, & Tjernberg, 2014). The observation of protein glycosylation in the extracted EPS raises interesting questions for future research: how much glycosylated proteins/polypeptides are present in the EPS? Are the proteins/polypeptides the same as the amyloid structure? What are the functions of those glycosylated proteins/polypeptides? Why there are different types of glycoconjugates?

#### 3.3.4 Intrinsic fluorescence of EPS

**Figure 7:**
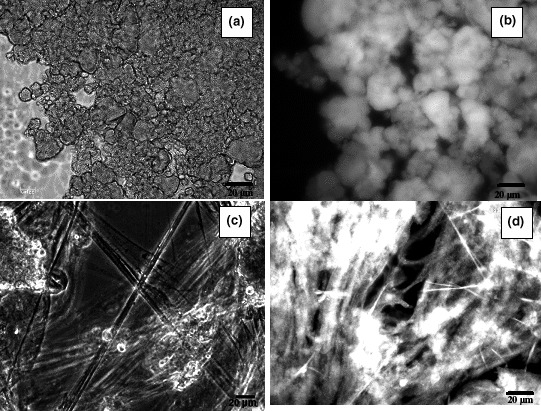
Autofluorescence of the nitrifying aerobic granular sludge and extracted EPS. (a) Light microscopy image of aerobic granular sludge; (b) autofluorescence image of aerobic granular sludge (excitation wavelength 436 nm and emission wavelength 477 nm); (c) light microscopy image of extracted EPS; (d) autofluorescence image of extracted EPS excitation (wavelength 436 nm and emission wavelength 477 nm)

Based on the above analysis, it is known that the extracted EPS is rich in cross‐β sheet structure, some of the proteins/polypeptides are glycosylated, and the EPS has a relatively high amount of tryptophan. Due to the fact that proteins are unique in displaying intense intrinsic fluorescence (autofluorescence) among biopolymers, and in proteins, tryptophan is the main fluorophore when the excitation wavelength is longer than 295 nm (Diaspro, 2010), it is assumed that the EPS might have autofluorescence properties when excited in a proper wavelength range. To test this hypothesis, confocal laser scanning microscopy (CLSM) was employed as a non‐destructive visualization method. When excited at around 436 nm wavelength, both the extracted EPS and the granules gave a strong emission image at around 477 nm (Figure 7). AOB microcolonies were clearly seen thanks to the autofluorescence. The EPS present fiber‐like morphology when precipitated out by ethanol. Those fibers give the same turquoise autofluorescence as the microcolonies do. This indicated that the EPS was extracted from AOB microcolonies.

Autofluorescence is an intrinsic property of proteins, if the autofluorescence of AOB microcolonies is comparable with that of the extracted EPS, it can be assumed that, AOB produces protein‐dominated EPS, the EPS fold to form a matrix to embed AOB in the microcolony. The FTIR spectroscopy showed that protein secondary structure of the extracted EPS was exactly the same as that of granules. Thus, heating the granules at 100°C with SDS likely only removed the quaternary or tertiary structures of proteins (which might be important to form AOB microcolony and the granule structure). It did not bring any change to the intrinsic property of the proteins.

#### 3.3.5 Mechanical strength of EPS

AOB are known to form relatively dense and strong microcolonies embedded in EPS (Liao, Allen, Leppard, Droppo, & Liss, 2002). As indicated by the autofluorescence study, the EPS was located in the AOB microcolonies. Rheological properties of the EPS may provide information on the role of EPS in providing the strength of microcolonies and granules.

**Figure 8:**
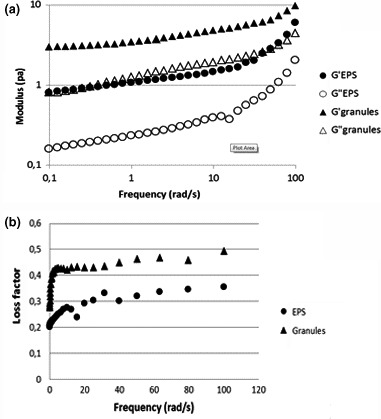
Rheological property of both nitrifying aerobic granular sludge and the extracted extracellular polymers (EPS)

The rheological behavior of both EPS and granules was investigated through strain sweeps and frequency sweeps. Strain sweep tests were performed mainly to determine the linear viscoelastic domain of the sample. In the frequency sweep tests, both the extracted EPS and granules display similar viscoelastic properties: G′ > G″ was observed over the entire frequency domain applied (Figure 8a). As G′ represents the solid‐like property and G″ represents the liquid‐like property, G′ > G″ means the solid property is more dominant than the liquid property. This also shows that the samples tested behave like a gel (Caggioni et al., 2007). Therefore, both the extracted EPS and granules have gel‐like structure with predominantly solid (elastic) properties. In addition, when the frequency increases, both the values of G′ and G″ and the slopes of the two curves increase (Figure 8a), indicating both the extracted EPS and the granules are mainly physically entangled/physically crosslinked (hydrogen bond or hydrophobic) systems. For a purely chemically cross‐linked gel, G′ and G″ would be independent of frequency; while for a physically entangled/crosslinked gel, the slope of G′ and G″ curves would increase with increased frequency (Grillet, Nicholas, & Lindsey, 2012).

Although both the extracted EPS and granules are physically entangled/crosslinked hydrogels, the stiffness of EPS hydrogel is different from that of granules. This can be clearly seen in Figure 8b. The ratio of G″/G′ is defined as tanδ, which is the “loss factor” (Mezger, 2014). It gives a measure of the viscous and the elastic portion of the viscoelastic deformation. It is shown in Figure 8b that the loss factor of the EPS increases at low frequency and remains constant at the frequency range between 30 and 100 rad/s. The increase indicates the hydrogel weakens at low frequencies, probably due to a gliding apart of the entangled structure and rearrangement. At high frequencies, there is no structure rearrangement anymore, the loss factor keeps constant. The same trend was observed for granules as well. The only difference is that the EPS hydrogel has lower loss factor than granules over the entire frequency range, indicating a stiffer hydrogel formed by EPS. Possibly, removing the quaternary or tertiary structures of proteins by SDS influence the stiffness.

To summarize, the extracted EPS keeps the physical structure of nitrifying aerobic granular sludge enriched with AOB. It is abundant with cross ß‐sheet proteins secondary structure, contains glycosylated proteins, rich in tryptophan and forms a hydrogel.

## 4 DISCUSSION

### 4.1 Amyloid‐like proteins in the structural EPS of aerobic granules enriched with AOB

The extracted structural EPS has amyloid like properties: rich in cross ß‐sheet and extremely resistant to chemical and thermal denaturation. Despite the fact that amyloids are commonly associated with human disease (e.g., Alzheimer's and Parkinson's disease), they are much more than just malicious entities (Dovidchenko, Leonova, & Galzitskaya, 2014). Nature has harnessed functional amyloids for beneficial use throughout all domains of life. Amyloids are found abundant in the EPS of a wide range of environmental and engineered biofilms, occupying 5%–40% of the biovolume (Larsen, Nielsen, Otzen, 2008; Larsen, Nielsen, Svendsen, 2008). Although the biological roles of amyloids in the EPS matrix is still poorly understood, it is proposed they increase the overall hydrophobicity of biofilms, increase their stiffness (Romero, Aguilar, Losick, & Kolter, 2010; Zeng et al., 2015) and even be an offensive EPS component such as an antimicrobial peptide (Destoumieus‐Garzón et al., 2003). In the current research, the extracted EPS is present in all AOB microcolonies, being a hydrogel matrix for the cells to be embedded in. It acts as a structural component that provides rigidity and keep the structure of both microcolonies and aerobic granules. Its comprehensive biological role remains to be determined.

Functional amyloids are detected in almost all environmental biofilms using amyloid‐specific dyes or antibodies (Dueholm & Nielsen, 2016). However, they have still only been purified and confirmed for a handful of pure cultured organisms (Larsen, Nielsen, Otzen, 2008). There is no report of their extraction from granular sludge. One of the reasons could be: the commonly used EPS extraction methodologies hinder functional amyloids’ discovery. Generally, harsh conditions are not recommended in EPS extraction since they cause cell lysis and structural change in the EPS (Flemming & Wingender, 2010). However, it is shown in the current research that, extracting at 100°C in 0.1% SDS for 30 min only leads to removing of protein quaternary and tertiary structures without causing any change in the secondary structure of the EPS proteins; even boiling in strong denaturing reagents (2M thiourea + 8M urea + 3% SDS) for 60 min can only disaggregate part of the proteins. Such chemically and thermally stable EPS is certainly impossible to be extracted by mild methods generally recommended in literatures, for example, centrifugation, sonication, use of cation exchange resins, addition of EDTA etc.

AOBs are known for forming strong microcolonies which are very resistant toward high shear forces and different physical/chemical manipulations (shear force of 2,000 S^−1^, addition of Triton X100, EDTA, increase pH to 9.5) (Larsen, Nielsen, Svendsen, 2008). Those microcolonies are considered as the non‐detachable fractions in the activated sludge flocs studied (Larsen, Nielsen, Svendsen, 2008). Due to the compactness and high adhesion characteristics of AOB microcolonies, EPS extracted from AOB microcolonies by conventional methods was always in relatively small amount (average of 20 mg BSA‐eq/g‐1 MLVSS, 9 mg glucose‐eq/g‐1 MLVSS) (Wang et al., 2014). In comparison, around 480 mg/g dry biomass (volatile fraction ratio) of EPS was extracted from AOB dominant aerobic granules by heating at 100°C in 0.1% SDS for 30 min in the current research. Furthermore, Curli associated Csg proteins were found present in *Nitrosomonas* marina (Data S1) (Curli is an amyloid produced by many enteric bacteria, e.g., *Escherichia coli* and *Salmonella* species). All these findings point to the possibility of amyloids production by AOBs as structural EPS. The reason why there is few report might be: most functional amyloids are remarkably stable. The conventional extraction methods could not solubilize those kind of EPS and separate them from the biomass. Therefore, effective amyloids extraction methodologies should be further developed.

### 4.2 Protein glycosylation in EPS

In the extracted EPS, some proteins, including the amyloid‐like proteins, are glycosylated with carbohydrates containing vicinal hydroxyl groups (e.g., neutral sugar), carboxyl (e.g., sialic acid) and sulfate ester groups.

Glycosylation of proteins is not only the most common but probably also the most important post‐translational modification process occurring in nature. 70% of proteins in eukaryotes are glycosylated. It is known to affect the expression, localization and life time of numerous proteins, which in turn, might be of relevance for protein function as well as downstream biological events such as the immune behavior of a cell (Valguarnera, Kinsella, & Feldman, 2016). Nowadays it is accepted knowledge that protein glycosylation systems are present in both eukaryotes and prokaryotes (Varki, Cummings, & Esko, 2015). Research concerning the functional importance of prokaryotic glycoconjugates is still lagging behind the wealth of information on eukaryotic studies. The field of prokaryotic glycosylation remains challenging due to its inherent complexity and enormous diversity. In several bacteria, glycosylation has been shown to impact protein function with regard to adhesiveness and invasiveness of host cells (Schäffer & Messner, 2017).

Glycosylation seems to make the proteins more chemical and thermal resistant. In the extracted EPS, the non‐glycosylated protein fragments were released after 10‐min denaturation pretreatment, while the glycosylated protein fragments were released only after 60‐min pretreatment. As the glycosylation in the bacterial system occurs on folded proteins, it was suggested that glycosylation acceptor site must be located in a flexible and surface‐exposed region of a folded protein (Kowarik et al., 2006), being involved in stabilizing the loop and reducing water activity, consequently making glycoprotein more stable than the non‐glycosylated protein in thermal denaturation (Min et al., 2009). Other functions of EPS glycosylation could be analogous to eukaryotic glycoprotein mucin. For example, Glycoconjugates containing neutral sugars protect the mucosa of fish against proteolytic degradation (Smith, 1989). Acid glycoconjugates are more defensive than neutral glycoconjugates against intestinal lumen contents (Smith, 1989). Sialic acid residues (rich in carboxyl groups) together with sulfate ester groups is responsible for the negative charge of the glycoconjugates and conceal receptor sites for viruses and mycoplasma species (Zimmer, Reuter, & Schauer, 1992). The presence of sulfate ester groups, deserves more attention. Such high acidity is essential to prevent the proliferation of pathogenic microorganisms (Zimmer et al., 1992). Moreover, the strong negative charge of sulfate ester groups may provide highly stable aqueous suspension as what is indicated by their function in stabilizing cellulose nano crystals from forming aggregation (Candanedo, Roman, & Gray, 2005). Therefore, the glycosylated proteins in the extracted EPS could have a similar function as mucin: maintenance of a hydrated layer, a barrier to pathogens and noxious substances and as a permeable gel layer for the exchange of gases and nutrients.

It is noted that components in EPS is generally defined as: polysaccharides, proteins, DNA, humic acids, etc. Protein and polysaccharides are quantified separately by colorimetric methods to get the relative quantity. The possibility of glycoproteins existence and the function of glycoproteins in the EPS is almost neglected. The abundance of glycosylated proteins in the extracted EPS strongly suggests that, glycoproteins might be one of the common components in the EPS of biofilm. The concept of EPS has to be redefined to include glycoproteins.

Glycosylation of amyloids has not been thoroughly investigated, although some correlations have been reported (Schedin‐Weiss et al., 2014). For example, an increase in the degree of sialylation of the glycans of amyloid precursor protein (APP) enhanced the secretion of both APP and its metabolites (Nakagawa et al., 2006). Obtaining the knowledge of amyloid glycosylation in biofilm systems is therefore important to generate a generic understanding of functional amyloids glycosylation. This knowledge may allow us to control amyloid formation in the future.

Overall, protein glycosylation is an energetically costly cellular process. Approximately 2% of the human genome encodes proteins involved in glycosylation events (Schäffer & Messner, 2017). Why AOBs produce glycosylated proteins in the EPS and how does it regulate are interesting topics for further research.

### 4.3 Function of tryptophan in the EPS

The extracted EPS has higher amount of tryptophan. Aromatic residues have critical structural roles in many β‐rich motifs and globular proteins (McGaughey, Gagne, & Rappe, 1998; Thomas, Meurisse, Charloteaus, & Brasseur, 2002). It was found that tryptophan residues promoted the aggregation of the amyloid fibers. It is likely that tryptophan triggers the aggregation process, stabilizes amyloidal structures with the bulky side chains to produce substantial interactions among adjacent residues (Biancalana et al., 2015). Even single tryptophan amino acids self‐associate into ordered superamolecular amyloid‐like fibrils was reported (Shaham‐Niv et al., 2017).

The role of tryptophan in biofilm formation was documented as well. Studies reported the induction of tryptophan biosynthesis genes during early biofilm formation in *E*. *coli* (Ren, Bedzyk, Thomas, Ye, & Wood, 2004; Domka et al., 2007). Tryptophan biosynthesis plays a role at the late stages of biofilm development in *Salmonella typhimurium*: in well‐established biofilm of *S*. *Typhimurium*, the biosynthetic genes of the trp operon were overexpressed (Yanofsky, 2001). These genes are required from the initial steps of tryptophan synthesis (i.e., from chorismate to indole) to the transfer of indole to tryptophan. In addition, there was a strong biofilm‐dependent induction of mtr, a tryptophan‐specific transporter. In this study, AOBs likely secret more tryptophan in the EPS to enhance aggregation and form a compact structure. Additional research has to be done to elucidate well the exact location and function of tryptophan in the EPS of granular sludge.

## CONFLICT OF INTEREST

The authors declare no conflict of interest.
