# Supplementary material for: Glycosylated amyloid‐like proteins in the structural extracellular polymers of aerobic granular sludge enriched with ammonium‐oxidizing bacteria
Source: Microbiologyopen. 2018 Mar 31;7(6):e00616. doi: 10.1002/mbo3.616 (PMC6291783; doi:10.1002/mbo3.616)
Supplement: Supplementary file 1 [file MBO3-7-e00616-s001.docx]

Supplementary information for the manuscript “Glycosylated amyloids in the structural extracellular polymers of aerobic granular sludge enriched with ammonium oxidizing bacteria”

**Table S1 Csg operon subunit in *Nitrosomonas marina***

| **Csg operon**  **subunit** | **Organism** | **Identity** | **Identity/Pos. %** |
| --- | --- | --- | --- |
| **E** | Nitrosomonas marina | Curli assembly protein, CsgE | 33/42 |
| **F** | Nitrosomonas marina | Curli production assembly/transport component CsgF | 37/44 |
| **G** | Nitrosomonas marina | Curli production assembly/transport component CsgG | 50/57 |

**Reactor operation**

The total volume of the granular airlift reactor was 5.2 L and the height-to-down comer diameter ratio was 16. The pH was measured online and automatically controlled at 8.0 ± 0.1 by the addition of a Na_2_CO_3_ 0.5 M solution. The temperature was maintained by using a cooling system and an electric heater connected to a temperature controller. A complete scheme of the reactor and more details about the operation can be found in Reino et al., (2016). Compressed air was supplied through an air diffuser placed at the bottom of the reactor and was manually manipulated to maintain the dissolved oxygen (DO) concentration in the bulk liquid in the desired value. Ammonium concentration in the bulk liquid was measured by using an on-line probe (AN-ISE sc probe with a Cartrical cartridge plus, Hach Lange, Germany) and was automatically controlled to maintain a fixed set point (32 mg N-NH_4_^+^ L^-1^) by varying the inflow rate by means of a proportional controller during the whole period of operation.

The reactor was fed with a synthetic influent with an average ammonium concentration of 70 mg N-NH_4_^+^ L^-1^. In addition, the synthetic wastewater contained 45 mg L^-1^ KH_2_PO_4_, 784 mg L^-1^ NaHCO_3_, 80 mg L^-1^ NaCl, 40 mg L^-1^ CaCl_2_, 90 mg L^-1^ MgCl_2_ and 1 mL of trace elements solution per L of influent consisting of 1.5 g L^-1^ FeCl_3_.6H_2_O, 0.18 g L^-1^ KI, 0.15 g L^-1^ CoCl_2_.6H_2_O, 0.12 g L^-1^ ZnSO_4_.7H_2_O, 0.12 g L^-1^ MnCl_2_.4H_2_O, 0.06 g L^-1^ Na_2_MoO_4_.2H_2_O, 0.03 g L^-1^ CuSO_4_.5H_2_O and 10 g L^-1^ EDTA.

The inoculum of the lab-scale airlift reactor was a granular nitrifying sludge from a pilot-scale reactor which performed partial nitritation of a real reject wastewater for more than 200 days (Torà et al., 2013) and was composed by 69 ± 9% of AOB and less than 1% of NOB (specifically *Nitrobacter* spp).

The continuous operation of the lab-scale airlift reactor performing stable partial nitritation of a low-strength synthetic influent from the inoculation until the stable operation at 10 °C was described in recent publications (Isanta et al., 2015; Reino et al., 2016). The continuous operation started after the inoculation at a temperature of 30 °C, being then gradually decreased until achieving stable partial nitritation at 20, 15, 12.5 and 10 °C. Table 1 shows the most relevant operational parameters obtained at the different temperatures used. Successful partial nitritation with efficient nitrite-oxidizing bacteria (NOB) repression was achieved during the whole operation by maintaining a low ratio between oxygen and ammonium concentrations in the reactor bulk liquid. Thus, DO/ N-NH_4_^+^ concentrations ratio was maintained at low values as it is shown in Table S2.

**Granular sludge and the dominant microorganisms**

The granular sludge was collected from the reactor, fixed by following the method described in Reino et al., (2016), and observed by using a scanning electron microscope (EVO MA10; Zeiss, Germany) at the following conditions: 20kv, 100pA, secondary electron detector (SE1).

Abundances of AOB and NOB were analyzed by FISH coupled to confocal laser scanning microscopy (CLSM) according to Reino et al., 2016. Single granules were washed several times in 1x PBS before being placed in the center of a cassette having biopsy pads on both sides. The cassette was completely submerged in formaldehyde 4% for fixing (24h). The cassette was embedded in paraffin wax before the sectioning with a microtome. The obtained slices (thickness of 3 µm) were placed on the surface of poly-L-lysine coated microscopic slides for the subsequent FISH hybridization. Specific probes for *Nitrosomonas spp*. (AOB) and *Nitrobacter spp*. (NOB) were 5’-6FAM-labeled and 5’-Cy3-labeled, respectively. Hybridizations were performed with the specific and general (5’-Cy5-labeled) probes described in Table S3 in Supporting Information. Image analyses were done with a Leica TCS-SP5 confocal laser scanning microscope (Leica Microsystem Heidelberg GmbH; Mannheim, Germany) using a Plan-Apochromatic 63x objective (NA 1.4, oil).

**Table S2.** Main operational parameters and results obtained in the operation of the lab-scale granular airlift reactor performing stable partial nitritation in the long-term. T: Temperature; NLR: Nitrogen Removal Rate; sAOR: Specific Ammonia Oxidation Rate; Nitrite/Ammonium: nitrite to ammonium concentrations ratio; DO: Dissolved Oxygen; DO/N-NH_4_^+^: dissolved oxygen to ammonium concentrations ratio.

| Days | T | NLR | sAOR | Nitrite/Ammonium | Nitrate produced | DO | DO/N-NH_4_^+^ |
| --- | --- | --- | --- | --- | --- | --- | --- |
|  | (°C) | (g N L^-1^ d^-1^) | (g N g^-1^ VSS d^-1^) |  | (mg N-NO_3_^-^ L^-1^) | (mg O_2_ L^-1^) | (mg O_2_ mg^-1^ N) |
| 36–76 | 20 | 1.2 ± 0.4 | 0.55 ± 0.04 | 1.1 ± 0.3 | 0.3 ± 0.1 | 3.2–5 | 0.11 ± 0.03 |
| 83–148 | 15 | 0.8 ± 0.2 | 0.32 ± 0.06 | 1.1 ± 0.2 | 0.4 ± 0.1 | 1.7–3.9 | 0.08 ± 0.02 |
| 149–482 | 12.5 | 0.7 ± 0.3 | 0.2 ± 0.1 | 1.2 ± 0.3 | 2.5 ± 0.7 | 0.4–3.4 | 0.06 ± 0.02 |
| 483–734 | 10 | 0.63 ± 0.06 | 0.18 ± 0.03 | 1.1 ± 0.2 | 0.6 ± 0.3 | 0.5–2.5 | 0.04 ± 0.02 |

**Table S3.** 16S rRNA-targeted oligonucleotide probes and target microorganisms to perform the fluorescence *in situ* hybridization analysis.

| Probe | Sequence (from ‘5 to ‘3) | Specificity |
| --- | --- | --- |
| EUB338 I | GCTGCCTCCCGTAGGAGT | Most bacteria |
| EUB338 II | GCTGCCTCCCGTAGGAGT | Planctomycetales |
| EUB338 III | CGCCATTGTATTACGTGTGA | Verrucomicrobiales |
| NSO190 | CGATCCCCTGCTTTTCTCC | β-AOB |
| NIT3 | CCTGTGCTCCATGCTCCG | *Nitrobacter* spp. |

Reference

Reino, C., Suárez-Ojeda, M.E., Pérez, J., and Carrera, J. (2016) Kinetic and microbiological characterization of aerobic granules performing partial nitritation of a low-strength wastewater at 10 °C. Water Res 101: 147–156.
